# A Case of Acute Liver Failure in a Patient on Isoniazid Prophylaxis for Latent Tuberculosis

**DOI:** 10.7759/cureus.22452

**Published:** 2022-02-21

**Authors:** Jobin Philipose, Kelly I Suchman, Danielle Aronsky, Tai-Ping Lee

**Affiliations:** 1 Gastroenterology and Hepatology, Staten Island University Hospital, Staten Island, USA; 2 Internal Medicine, Northwell Health, Manhasset, USA; 3 Internal Medicine, Northwell Health, Long Island Jewish Medical Center, New Hyde Park, USA; 4 Hepatology, Northwell Health, Long Island Jewish Medical Center, New Hyde Park, USA

**Keywords:** antituberculosis treatment, latent tuberculosis treatment, severe hepatotoxicity, drug-induced acute liver failure, isoniazid

## Abstract

Isoniazid (INH) is widely used for latent *Mycobacterium tuberculosis* despite the known risk of liver injury, with severe hepatitis occurring in up to 1% of patients. We report a patient who presented with two weeks of anorexia, nausea, and jaundice following six months of INH monotherapy for latent tuberculosis (TB). After other causes of liver injury were ruled out, she underwent a liver biopsy showing submassive necrosis, hepatocellular dropout, and lobular inflammation with no evidence of fibrosis. She was also found to have acute portal hypertension. She was diagnosed with drug-induced liver injury (DILI) and was treated with n-acetyl cysteine (NAC), ursodiol, and vitamin K. She recovered without the need for a liver transplant. This case supports the need for monitoring of liver tests in high-risk individuals on INH therapy to reduce the risk of hepatotoxicity.

## Introduction

Latent tuberculosis (TB) affects around nine million (3%) Americans [1]. Isoniazid (INH) therapy has been considered the standard of care for latent *Mycobacterium tuberculosis* for decades [2]. Although known to cause liver injury, INH continues to be widely used due to its efficacy. Up to 20% of patients taking INH experience asymptomatic increases in aminotransferase levels. Further, severe hepatitis occurs in up to 1% of patients [3], although only a few case reports describing this have been published [4]. Herein, we report a patient on INH prophylaxis developing subacute liver failure with concurrent portal hypertension in the absence of chronic liver disease who recovered without a liver transplant. Therefore, risk stratification for monitoring liver tests and raising awareness of symptoms and signs of INH hepatotoxicity are warranted to prevent its morbidity and mortality.

## Case presentation

A previously healthy 44-year-old female with gastroesophageal reflux disease on pantoprazole and latent TB on INH for six months was referred to the emergency room by her primary care physician for new-onset elevated liver tests. She is a healthcare aid and immigrated from Bangladesh three years ago. Her liver tests prior to starting INH were within normal limits.

She developed right-sided abdominal pain, anorexia, and jaundice about two weeks prior to the emergency room visit. INH was stopped as the symptoms began two weeks ago. She denied any use of illicit drugs, alcohol, herbal supplements, over-the-counter medications, occupational exposures, or smoking. She also denied any known family history of liver or autoimmune diseases.

On presentation, she was tachycardic and had altered mental status with asterixis. Blood tests showed an alanine aminotransferase (ALT) of 651 U/L (normal range: 4-33 U/L), aspartate transaminase (AST) of 1,134 U/L (normal range: 4-32 U/L), alkaline phosphatase (ALP) of 186 U/L (normal range: 40-120 U/L), total bilirubin (TB) of 14.1 mg/dL (normal range: 0.2-1.2 mg/dL) with direct bilirubin (DB) of 7.7 mg/dL (normal range: 0.0-0.2 mg/dL), international normalized ratio (INR) of 1.75 (normal range: 0.88-1.16), prothrombin time (PT) of 19.6 seconds (normal range: 10.6-13.6 seconds), activated partial thromboplastin time (APTT) of 42.7 seconds (normal range: 27.0-36.3 seconds), albumin of 3.2 g/dL (normal range: 3.3-5.0 g/dL), sodium (Na) of 136 mmol/L (normal range: 135-145 mmol/L), platelets of 204 K/uL (normal range: 150-400 K/uL), and normal lipase of 64 U/L (normal range: 7-60 U/L). The model for end-stage liver disease (MELD)-Na score was 24 (Table 1).

**Table 1:** Patient laboratory values. TB, total bilirubin; DB, direct bilirubin; ALT, alanine aminotransferase; AST, aspartate aminotransferase; ALP, alkaline phosphatase; INR, international normalized ratio; PT, prothrombin time; APTT, activated partial thromboplastin time; Na, sodium; MELD, model for end-stage liver disease.

	TB (mg/dL)	DB (mg/dL)	ALT (U/L)	AST (U/L)	ALP (U/L)	Platelets (K/uL)	INR	PT (seconds)	APTT (seconds)	Albumin (g/dL)	Na (mmol/L)	Creatinine (mg/dL)	MELD-Na
Reference values	0.2–1.2	0–0.3	0–41	0–41	30–115	130–400	0.6–1.3	9.95–12.87	27-39.2	3.5–5.2	135–146	0.7–1.5	>6
Day 1	14.1	7.7	651	1,134	186	204	1.75	19.6	42.7	3.2	136	0.63	24
Day 2	13.2		591	1,013	179	200	1.86	20.8	37.8	2.8	136	0.59	25
Day 5	11.1		440	736	147	149	1.93	21.5	40.1	2.2	139	0.65	23
Day 9	14.7	9.8	380	518	99	117	3.14	34.2	43.0	2.4	131	0.57	32
Day 12	9.0		159	114	107	112	2.16	23.7	36.5	1.9	134	0.60	26
Day 19	6.3		62	120	113	187	1.7	19.1	40.9	2.2	139	0.60	20
Day 45	3.2		63	110	137	225	1.1	11.1	28	3.2	138	0.64	13

An abdominal ultrasonogram with Doppler on admission showed mild subcapsular nodularity in the liver, small ascites, no splenomegaly, and a normal biliary tree with no vascular abnormality. Workup for metabolic, viral, and immune-mediated liver diseases was negative, including hepatitis A virus (HAV) immunoglobulin (Ig) M/IgG, HBsAg, hepatitis B surface antigen (HBsAb), hepatitis B core antibody (HBcAb) IgM/IgG, hepatitis C virus (HCV) Ab, anti-hepatitis E virus (HEV), serum ferritin, transferrin saturation, ceruloplasmin level, ANA, SMA, immunoglobulin panel, AMA, anti-LK microsomal Ab, anti-soluble liver antigen (Ag), EBV, CMV, and herpes serology. The Roussel Uclaf Causality Assessment Method (RUCAM) score was 8 (indicating probable hepatotoxicity), and the R ratio was 17 on admission (hepatocellular drug-induced liver injury (DILI)). The patient was subsequently diagnosed with INH-induced acute liver failure.

**Figure 1:**
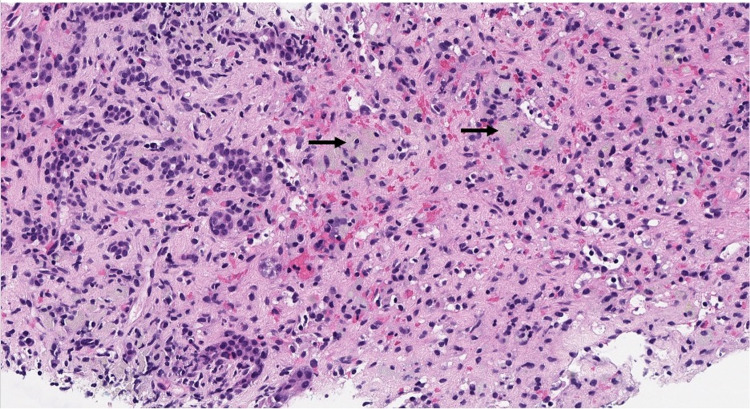
Bile ductular proliferation and hepatocellular collapse with ceroid-laden macrophages (arrows) (20× H&E).

**Figure 2:**
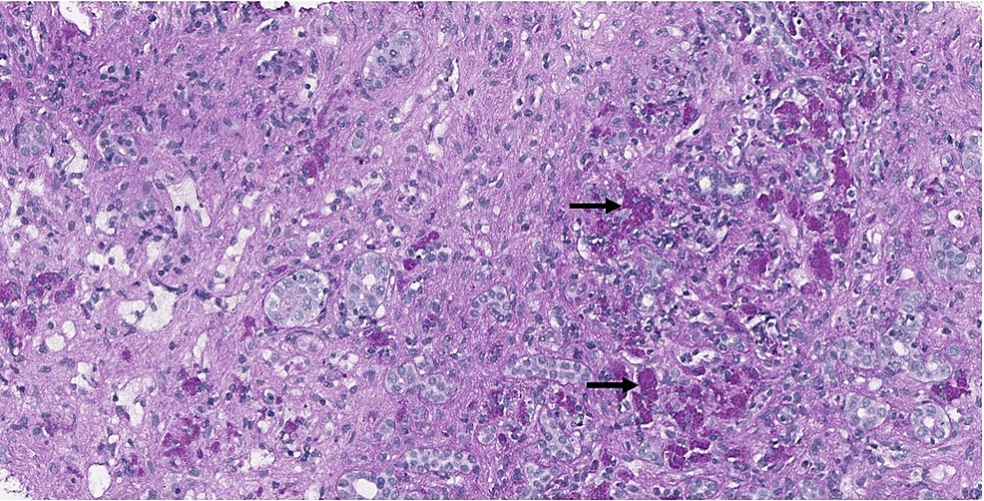
Ceroid-laden macrophages (arrows) highlighted by PAS stain after diastase (20× PAS/D).

**Figure 3:**
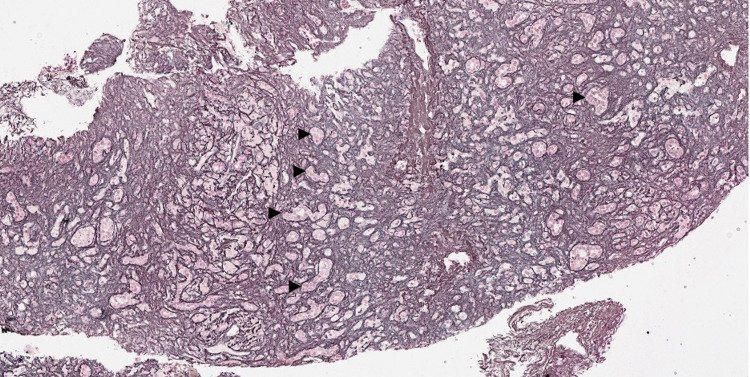
Reticulin stain demonstrating hepatocellular collapse and prominent bile ductular proliferation (arrowheads) (20× reticulin).

**Figure 4:**
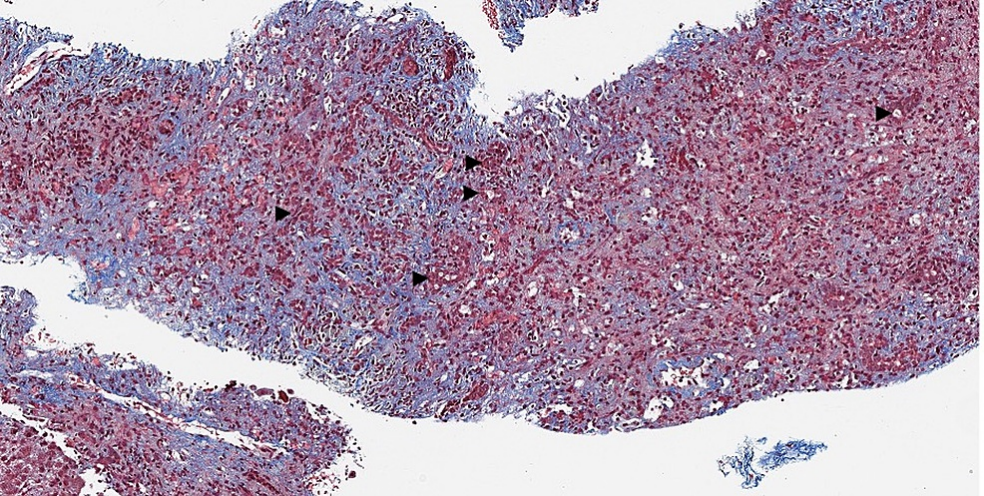
Trichrome stains demonstrating hepatocellular collapse (faint blue staining) and prominent bile ductular proliferation (arrowheads) (20× trichrome).

She was started on a 21-hour intravenous n-acetyl cysteine (NAC) protocol for non-acetaminophen drug-induced liver injury. A magnetic resonance imaging (MRI) with contrast and magnetic resonance cholangiopancreatography (MRCP) of the abdomen five days after admission reported mild nodularity of the hepatic contour and increasing abdominal ascites. She underwent a transjugular (TJ) liver biopsy four days later due to clinical deterioration with worsening jaundice and INR. Liver biopsy demonstrated marked submassive necrosis, hepatocellular dropout, and lobular inflammation with no evidence of fibrosis, a pattern consistent with acute cholestatic injury most compatible with a clinical diagnosis of drug-induced liver injury (Figures 1-4).

The patient began to experience worsening abdominal pain, as well as leukocytosis to 14,000 K/uL with 25.5% bands three days post-TJ liver biopsy. She completed another 21-hour NAC infusion. She was then started on meropenem for the empiric coverage of suspected infection. Workup for infection, including blood cultures, urine tests, chest radiography, and contrast abdominal computed tomography scan to rule out intrahepatic or intra-abdominal hemorrhage or abscess, was negative. A subsequent paracentesis revealed a serum ascites albumin gradient of 1.2 consistent with portal hypertension and no spontaneous bacterial peritonitis. She was started on salt restriction, oral furosemide 40 mg daily for ascites and leg swelling, ursodiol 500 mg twice daily for cholestasis, and vitamin K 5 mg for three days.

Her abdominal distension decreased, and hepatic encephalopathy resolved. Her liver enzymes, along with INR, continued to downtrend, as summarized in Table 1. At outpatient follow-up one month later, the patient was asymptomatic, with resolution of pedal edema, and furosemide was discontinued. On repeat bloodwork, her liver enzymes remained slightly elevated, as shown in Table 1. She is advised to continue close follow-up.

## Discussion

Drug-induced liver injury (DILI) is the most common cause of acute liver failure in the United States and is a diagnosis of exclusion. To diagnose DILI, chemistries must meet one of the following criteria: ALT greater than or equal to five times the upper limit of normal (ULN), ALP greater than or equal to two times ULN, or ALT greater than or equal to three times ULN with simultaneous elevation in bilirubin greater than two times ULN. Isoniazid is considered an idiosyncratic cause of DILI, which is not dose-dependent. The mechanism of isoniazid-induced DILI is not well elucidated. Hepatotoxicity likely results from direct toxicity of free radicals generated by the drug's metabolites or secondary to an immune response. Isoniazid also inhibits the production of several cytochrome p450 enzymes, increasing the hepatotoxicity of other drugs such as rifampin and itself. As such, liver injury is seen with the combination of rifampin and INH more often than with either drug alone. No evidence currently exists for a synergistic effect between pantoprazole and INH causing DILI.

INH-induced liver injury has a myriad of presentations. Mild (aminotransferase levels < 100 U/L) subclinical liver injury appears in up to 20% of patients taking INH. If the presentation is mild, the drug can be continued with close monitoring, and liver tests typically normalize. However, severe hepatitis can occur in up to 1% of exposed patients [3]. The common clinical presentations include fatigue, malaise, and nausea. Jaundice is the presenting symptom in 10% of these cases. Most cases occur within 2-3 months of starting therapy, although reported cases range from one week to 14 months.

Several patient characteristics have been found to increase susceptibility to isoniazid hepatotoxicity, including the history of alcoholism, viral hepatitis, advanced age, Asian racial background, slow acetylator status, and female sex.

The idiosyncratic nature of isoniazid DILI makes the clinical course and outcomes of patients with the disease difficult to predict. In addition to stopping the medication, our patient was treated medically without the need for an orthotopic liver transplant, which was reported in some cases with acute liver failure [4]. Besides stopping the suspected agent, no definitive therapies exist for idiosyncratic DILI. Guidelines suggest consideration of NAC therapy, and few studies exist regarding the benefits of corticosteroid therapy. Furthermore, our patient's acute liver failure had concurrent portal hypertension, an association that occurs in acute liver failure. Her liver enzymes remained elevated at a follow-up visit one month later, and she will continue to be followed closely. It remains unclear if she may progress to chronic liver injury (defined as elevated liver tests greater than six months from onset) [5]. In terms of retreatment of latent tuberculosis infection as per the American Thoracic Society (ATS), INH should be stopped permanently if ALT is more than five times the upper limit of normal, and alternatives such as rifampin for four months can be considered, although some experts recommend that further treatment should be carefully made on a case-by-case basis, weighing the risk of progression to TB disease against the risk of rifampin-induced DILI.

## Conclusions

Despite known INH hepatotoxicity, there are no standard guidelines for monitoring liver tests. The CDC does not recommend routine testing of hepatic function unless the patient has baseline abnormalities or is at increased risk of hepatotoxicity. The ATS suggests that baseline and monthly follow-up liver tests be obtained in high-risk individuals (including those with chronic liver disorders, chronic alcohol use, HIV on HAART therapy, and pregnant women), as well as individuals over the age of 35 on an individualized basis. Guidelines also state that isoniazid therapy should be stopped if the ALT is >3 times the upper limit of normal when jaundice or other hepatotoxic symptoms are present or five times the upper limit in the absence of symptoms.
